# Capsaicin-Sensitive Peptidergic Sensory Nerves Are Anti-Inflammatory Gatekeepers in the Hyperacute Phase of a Mouse Rheumatoid Arthritis Model

**DOI:** 10.3390/ijms22041682

**Published:** 2021-02-08

**Authors:** Bálint Botz, Gábor Kriszta, Kata Bölcskei, Ádám István Horváth, Attila Mócsai, Zsuzsanna Helyes

**Affiliations:** 1 Department of Medical Imaging, Medical School, University of Pécs, H-7624 Pécs, Hungary; 2 János Szentágothai Research Centre, Molecular Pharmacology Research Team, University of Pécs, H-7624 Pécs, Hungary; gabor.kriszta@aok.pte.hu (G.K.); kata.bolcskei@aok.pte.hu (K.B.); adam.horvath@aok.pte.hu (Á.I.H.); 3 János Szentágothai Research Centre, NMR Core Facility, University of Pécs, H-7624 Pécs, Hungary; 4 Department of Pharmacology and Pharmacotherapy, Medical School, University of Pécs, H-7624 Pécs, Hungary; 5 Department of Physiology, Semmelweis University, H-1094 Budapest, Hungary; mocsai.attila@med.semmelweis-univ.hu; 6 PharmInVivo Ltd., H-7629 Pécs, Hungary

**Keywords:** sensory neuropeptides, inflammation, neurogenic inflammation, arthritis, novel drug targets

## Abstract

Capsaicin-sensitive peptidergic sensory nerves play complex, mainly protective regulatory roles in the inflammatory cascade of the joints via neuropeptide mediators, but the mechanisms of the hyperacute arthritis phase has not been investigated. Therefore, we studied the involvement of these afferents in the early, “black box” period of a rheumatoid arthritis (RA) mouse model. Capsaicin-sensitive fibres were defunctionalized by pretreatment with the ultrapotent capsaicin analog resiniferatoxin and arthritis was induced by K/BxN arthritogenic serum. Disease severity was assessed by clinical scoring, reactive oxygen species (ROS) burst by chemiluminescent, vascular permeability by fluorescent in vivo imaging. Contrast-enhanced magnetic resonance imaging was used to correlate the functional and morphological changes. After sensory desensitization, both early phase ROS-burst and vascular leakage were significantly enhanced, which was later followed by the increased clinical severity scores. Furthermore, the early vascular leakage and ROS-burst were found to be good predictors of later arthritis severity. We conclude that the anti-inflammatory role of peptidergic afferents depends on their activity in the hyperacute phase, characterized by decreased cellular and vascular inflammatory components presumably via anti-inflammatory neuropeptide release. Therefore, these fibres might serve as important gatekeepers in RA.

## 1. Introduction

Rheumatoid arthritis (RA) is globally the most common autoimmune joint disease, thus it represents a considerable burden for both the patients and the society. The last decades have seen tremendous improvement in the management of RA due to major advances in its biological therapy (e.g., monoclonal antibodies) [1]. Nonetheless, chronic sequelae of RA such as joint pain remain to be challenging to manage. Hence, it is important to better understand the pathophysiological underpinnings of the neurogenic factors involved in the inflammatory cascade, in order to identify the novel pathways for therapeutic interventions. 

Capsaicin-sensitive afferents are abundant on the periphery, they densely innervate the joint capsule and synovial tissue. These sensory nerves are not only important for pain perception, but also exert efferent functions via the local release of a plethora of mainly peptide mediators [2,3,4]. Some of these sensory neuropeptides are primarily algogenic and proinflammatory, while others are anti-inflammatory and analgesic. Thus, the net effect is variable and dependent on the proportion of different mediators and their roles in the complexity of the pathophysiological cascade. A characteristic feature of capsaicin-sensitive afferents is the expression of transient receptor potential vanilloid 1 (TRPV1) and ankyrin 1 (TRPA1) receptors. These receptors can be activated by a variety of endo-, and exogenous substances including capsaicin, resiniferatoxin (RTX), protons (in case of tissue acidosis), and a diverse array of proinflammatory mediators [5,6]. Receptor activation leads to the release of proinflammatory neuropeptides such as the tachykinin family, calcitonin gene-related peptide (CGRP), vasoactive intestinal polypeptide (VIP), and pituitary adenylate cyclase-activating polypeptide (PACAP). These mediators induce vasodilation, plasma protein extravasation, and early inflammatory cell activation, collectively called neurogenic inflammation [7]. However, anti-inflammatory mediators, such as somatostatin or galanin can also be released [8]. It has been shown using gene-deficient mice that selective deletion of the genes of such peptides can alter disease severity in mouse models of RA [9,10]. 

The first sporadic clinical data about neurogenic factors playing a significant role in the development of autoimmune joint disorders were published nearly a century ago. Since then it has become established that neurogenic pathways play a pivotal role not only in inflammation, but also in the normal homeostatic function of the joints [11,12]. It has been confirmed, that there is a bidirectional communication between peripheral nociceptors and immune cells via their released mediators [13,14]. Clinical evidence gathered in the recent decades also indicates that altered levels of these neuropeptides in RA patients have a profound influence on disease severity [15,16,17,18,19], but our understanding of the mechanisms responsible for this effect is still incomplete.

Therefore, in the present experiment we used the K/BxN serum transfer mechanism model of RA and investigated the role of capsaicin-sensitive sensory nerves in vascular and cellular components of this condition. This model is induced by transferring the autoantibody-rich serum of the transgenic K/BxN mice into naïve recipients, where it elicits a robust polyarthritis. The inflammation is mainly caused by the autoantibodies against the ubiquitously expressed glucose-6-phosphate isomerase (G6PI) self-antigen, which elicits a robust activation of the innate immune system [20]. As the K/BxN serum transfer model relies on passive transfer of autoantibody-rich serum into recipient mice, it triggers autoimmune joint inflammation with nearly 100% penetration in a variety of susceptible mouse strains. We previously showed that selective desensitization of these nerve terminals leads to increased disease severity in multiple models of RA [9,21,22,23,24]. In the K/BxN serum transfer model increased disease severity measured by hind limb volume and clinical severity scoring was found, coupled with diminished mechanical hyperalgesia in desensitized mice. Prior results thus indicate, that while capsaicin-sensitive afferents are crucial to inflammatory hyperalgesia, they also possess important anti-inflammatory properties on the periphery. However in the earlier studies the primary focus was on the acute to chronic phases of the disease, while it has been shown that in the K/BxN model subtle, primarily microvascular changes (vasodilation and increased permeability facilitating edema formation and leukocyte transmigration) occur within minutes to hours, and predate any functional or morphological changes, which typically take at least a day to develop [25]. As neurogenic inflammation is heavily dependent on microvascular response, in this study we aimed to shift the focus toward this very early “hyperacute” phase of the model, where functional or morphological changes are nonexistent. Recent developments in the field of in vivo optical imaging have also shown that the extremely weak photonic radiation caused by the decay of free radicals such as singlet oxygen can be imaged without utilizing exogenously administered imaging probes in inflammatory arthritis, albeit their detection remains challenging [26,27]. Thus, in this study we also aimed to determine whether with a dedicated imaging sequence having increased sensitivity this phenomenon is within the grasp of commercially available imaging systems. 

**Figure 1:**
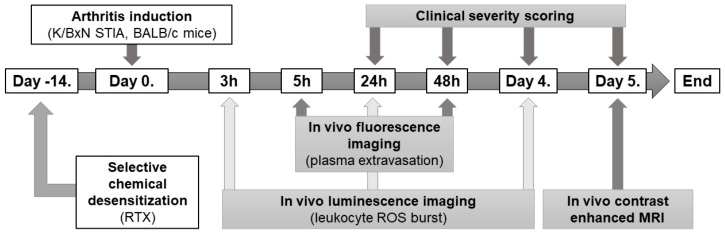
Flowchart summary of the experimental protocol.

Altogether, in this study we therefore aimed to assess the role of capsaicin-sensitive afferents in a mouse model of the K/BxN serum transfer arthritis, using in vivo optical imaging to measure the inflammatory vascular leakage and reactive oxygen species (ROS) burst due to leukocyte activation in the hyperacute phase of the disease. Functional inflammatory changes such as edema and erythema were assessed by semiquantitative clinical scoring. For better evaluation of the morphological parameters in vivo contrast enhanced magnetic resonance imaging (MRI) of the inflamed joints was also performed. The experimental layout has been summarized in Figure 1.

## 2. Results

### 2.1. Increased Arthritis Severity after Sensory Defunctionalization

**Figure 2:**
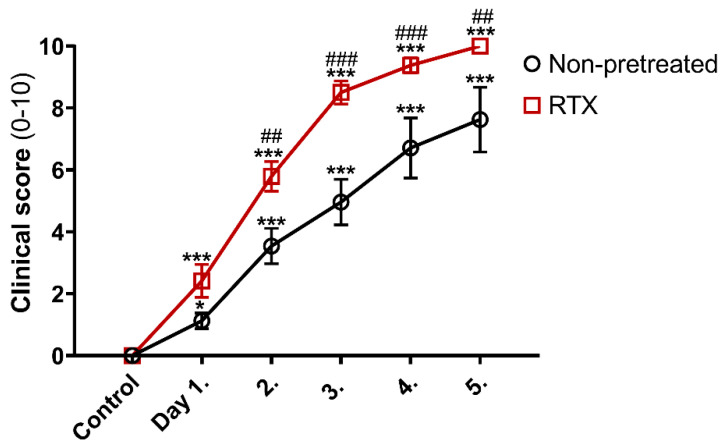
Clinical arthritis severity scores of non-desensitized and RTX-desensitized (sensory defunctionalized) mice. Data points represent mean values ± SEM of *n* = 6/group, both hindlimbs; two-way ANOVA and Fishers LSD test, * *p* < 0.05, *** *p* < 0.001 vs. pre-treatment self-control values, ## *p* < 0.01, ### *p* < 0.001 vs. non-pretreated group.

Significant tibiotarsal joint edema and hindpaw hyperemia developed on day 1 in both the non-desensitized and RTX-desensitized K/BxN serum-treated arthritic groups. It remained significantly greater in RTX-pretreated mice compared to their non-desensitized controls from day 2 onwards till the end of the experiment, disease severity peaking by day 5 (Figure 2). 

### 2.2. Facilitated Early Arthritic Vascular Hyperpermeability after Sensory Defunctionalization

**Figure 3:**
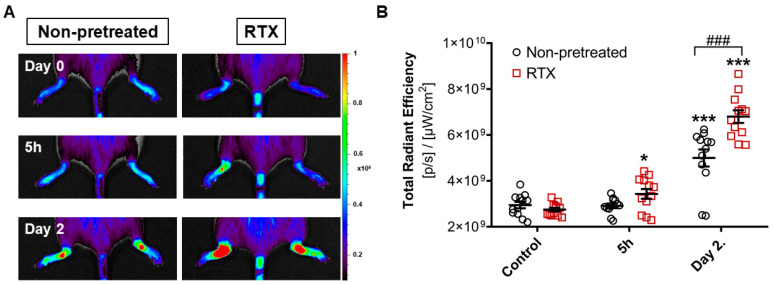
Inflammatory vascular hyperpermeability measured by in vivo fluorescence imaging and a vascular leakage probe. (**A**) Representative images. (**B**) Fluorescence signal of the ankle joints. Individual values are shown together with the means ± SEM of *n* = 6 per group both hindlimbs; two-way ANOVA and Fishers LSD test, * *p* < 0.05, *** *p* < 0.001 vs. self-control values before arthritis induction, ### *p* < 0.001 vs. non-pretreated arthritic group.

Fluorescent tracer accumulation reflecting vascular permeability and plasma leakage was significantly increased in RTX-desensitized mice, but not in the non-desensitized arthritic group as early as 5 h after K/BxN serum transfer. On day 2 contrast agent extravasation was robustly increased in both groups compared to the pretreatment baseline value, while still being significantly greater in the desensitized animals (Figure 3A,B). 

### 2.3. Increased Early-Phase Arthritic Free Radical Production after Sensory Defunctionalization

**Figure 4:**
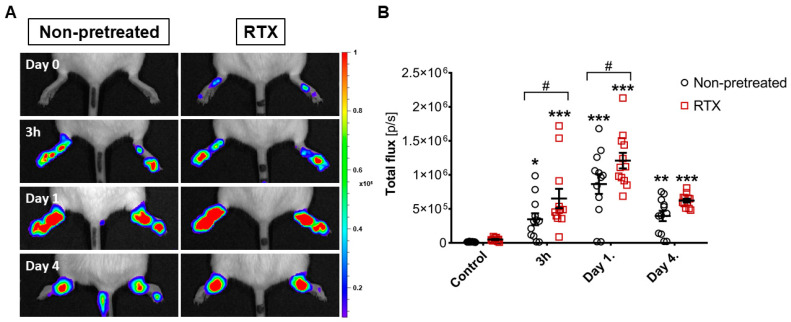
Inflammatory reactive oxygen species production of leukocytes assessed by in vivo luminescence imaging and the free radical sensor L-012. (**A**) Representative images and (**B**) Luminescence signal of the ankle joints. Individual values, means, ± SEM, two-way ANOVA and Fishers LSD test, *n* = 6/group, both limbs. * *p* < 0.05, ** *p* < 0.01, *** *p* < 0.001 vs. self-control values before arthritis induction, # *p* < 0.05 vs. non-pretreated arthritic group.

Already at 3 h following K/BxN serum challenge both RTX-desensitized and non-desensitized arthritic groups demonstrated significantly increased luminescence signal indicating elevated free radical production. Both at 3 h and day 1 the luminescence signal was significantly greater in the hindlimbs of RTX-pretreated mice. Later the signal became more circumscribed to the ankle joints and also subsided in both groups by day 4 (Figure 4A,B). 

**Figure 5:**
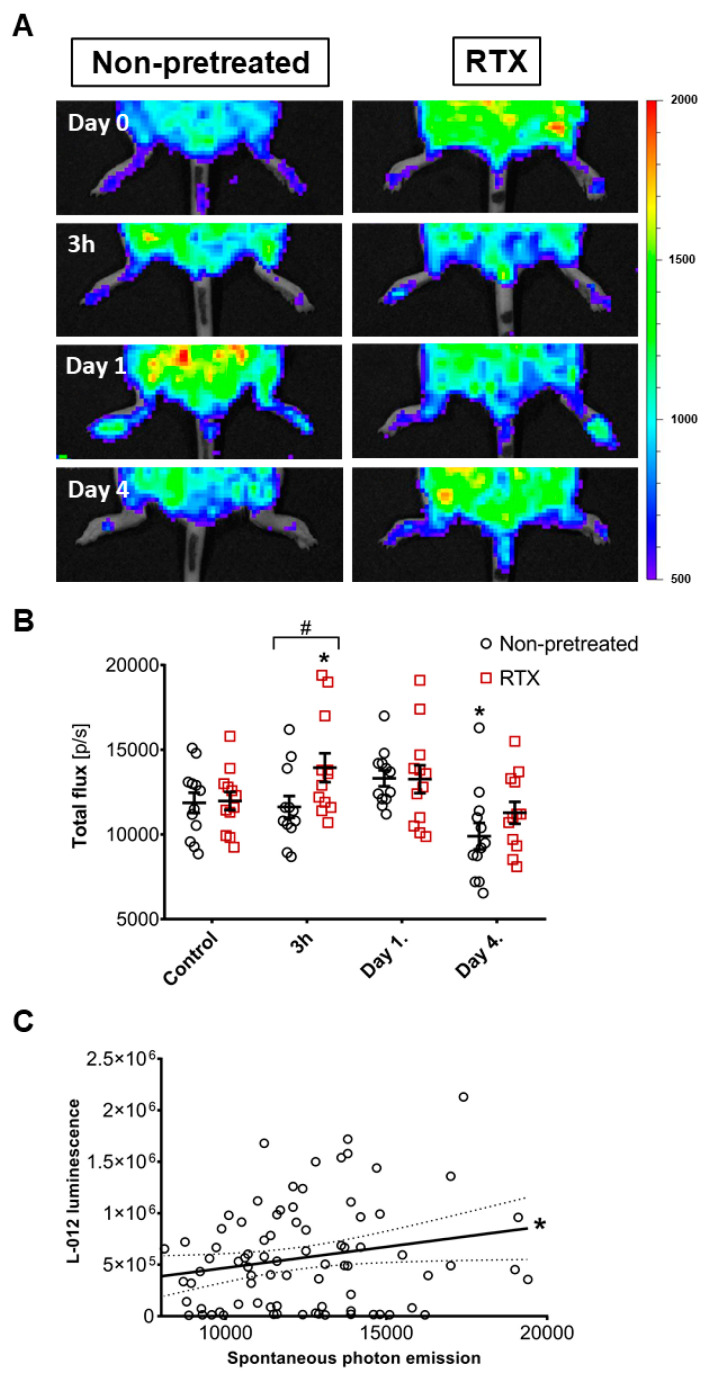
In vivo spontaneous ROS-derived photon emission. (**A**) Representative luminescence images, (**B**) Quantitative data. Individual values, means, ± SEM, two-way ANOVA and Fishers LSD test, *n* = 6/group, both hindlimbs. * *p* < 0.05, vs. self-control values before arthritis induction, # *p* < 0.05 vs. non-pretreated arthritic group. (**C**) Linear regression and correlation of the spontaneous photon signal and the L-012-derived luminescence. All matched data pairs shown from both non-pretreated and RTX-pretreated groups. * *p* < 0.05.

Spontaneous luminescence signals were similar in both groups prior to the induction of inflammatory arthritis. 3 h after the transfer of the autoantibody-rich serum, a modest but significant increase was observed in the RTX-pretreated mice compared to both their own pre-challenge values and the non-pretreated ones (Figure 5A,B). Despite the increasing clinical severity this photon emission returned to pre-challenge levels in RTX-pretreated mice, and in the non-pretreated group it became even lower than the initial self-control values. To assess how well spontaneous photon emission and L-012 derived luminescence signal correlates the paired values of these measurements were plotted against each other, showing a weak but significant correlation between these parameters (Figure 5C). 

### 2.4. Early Vascular Hyperpermeability and ROS Production Are Good Predictors of Later Disease Severity, and Correlate well with Free Radical Production

**Figure 6:**
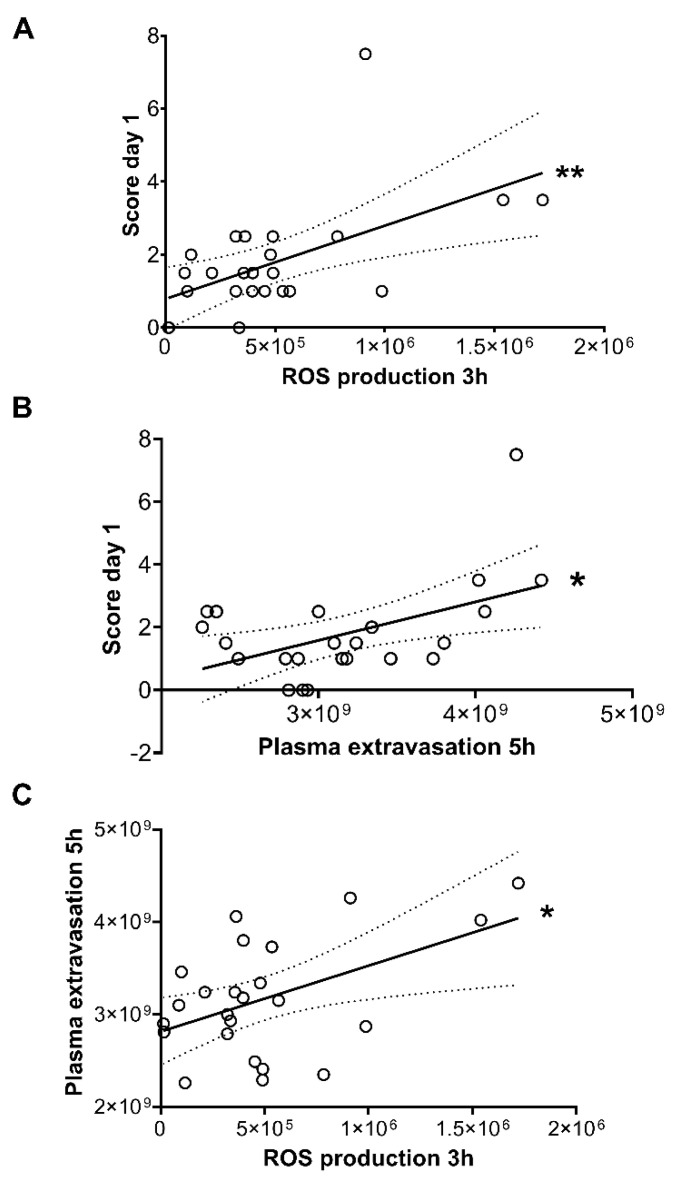
Linear regression and correlation of (**A**) L-012 luminescence signal at 3 h and the clinical severity score on day 1. (**B**) Fluorescent vascular signal at 5 h and the day 1 score, (**C**) the vascular fluorescent signal and the L-012-derived luminescence. All matched data pairs shown from both non-pretreated and RTX-pretreated groups. * *p* < 0.05, ** *p* < 0.01.

We also compared the semiquantitative clinical arthritis severity scores on day 1 with both the luminescent ROS production signal at 3 h and the vascular leakage fluorescent signal at 5 h both in the RTX-desensitized and non-desensitized groups. A significant trend was observed indicating that the early-phase plasma leakage correlates well with the macroscopic changes reflected by the clinical severity score, which emerges only later (Figure 6A,B). Comparison of the early plasma leakage and free radical production also showed a positive correlation (Figure 6C). 

### 2.5. Synovial Enhancement in the Contrast-Enhanced Ankle Joint MRI 

**Figure 7:**
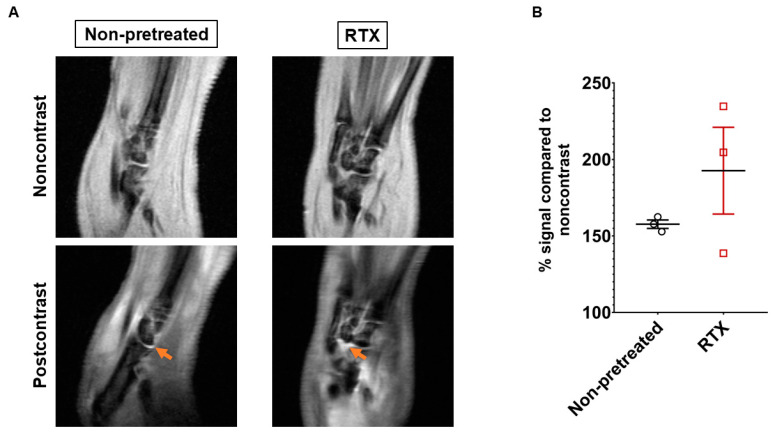
In vivo contrast enhanced T1-weighted MRI of the inflamed ankle joints on day 5. (**A**) Representative images pre- and postcontrast (iv. gadolinium). Increased synovial signal highlighted by arrows on the postcontrast images. (**B**) Quantitative evaluation of intraarticular signal intensity after the contrast agent (*n* = 3/group right hindlimb, unpaired *t*-test).

The soft tissue edema was well demonstrated by the MRI in line with macroscopic observations. On the postcontrast acquisition the greatest enhancement was observed in and around the ankle and smaller tarsal joints, suggesting synovial and intra-articular accumulation of the contrast agent (Figure 7A). The semiquantitative evaluation showed increasing tendency of the joint signal in RTX-pretreated mice. However, due to the low throughput of the technique, and the small number of imaged subjects, no statistically significant difference could be determined between the RTX-desensitized and non-desensitized groups (Figure 7B).

## 3. Discussion

This study demonstrates that the protective, anti-inflammatory effect of capsaicin-sensitive afferents predominantly manifests in the early, hyperacute phase of the K/BxN serum transfer model of RA. This is largely due to diminished vasodilation and vascular dysregulation following the autoantibody-challenge, as well as in reduced early ROS-burst and leukocyte activation. This results in a decreased clinical severity later. The MRI imaging showed that persistent inflammatory hyperenhancement in the mature phase of the model is predominantly localized to the synovial tissues. These findings support earlier results demonstrating increased vascular permeability in the same model after sensory defunctionalization, which affects the sites that later develop clinically apparent arthritis [28]. The synovial tissue is particularly highly vascularized, and also densely innervated by both myelinated and unmyelinated nerve fibers [29]. This is also supported by the results of the MRI imaging in our experiment, showing increased synovial enhancement post iv. contrast agent. These findings altogether suggest that early synovial vascular leakiness and consequent leukocyte extravasation are controlled by locally released mediators from the capsaicin-sensitive peptidergic afferents. 

We previously showed in the K/BxN serum transfer, proteoglycan-induced autoimmune arthritis, and complete Freund’s adjuvant-induced chronic arthritis models that desensitization of the capsaicin-sensitive afferents results in increased disease severity and inflammatory cell activity, coupled with diminished hyperalgesia [21,22,23]. However, in prior studies the experimental layout way conceived based on the course of clinical disease severity and functional changes. These results demonstrate that robust vascular and cellular changes predate functional and morphological impairment, which should be reflected by experimental protocols incorporation in in vivo imaging. Thus, imaging in the clinically elusive, “black box” phase of the disease has a high potential to better understand the underpinnings of functional and morphological arthritic changes. 

It should be emphasized that the role of capsaicin-sensitive afferents is not ubiquitously beneficial and depends on the pathophysiological mechanisms. We previously showed that disease severity in e.g., the collagen antibody-induced RA model (CAIA) [24] and inflammatory changes in the mast cell tryptase-induced acute arthritis model are diminished after RTX-pretreatment [30]. This highlights the challenge posed by the various RA models, which typically only mimic certain aspects of the human disease maintained by multiple autoantibodies and a complex underlying dysregulation of the immune system. Furthermore, it also underlies that models dependent on a single aspect of the diseases (e.g., mast cell activation, or a single autoantibody) do not necessarily address well the role of the investigated pathway in a complex clinical condition. Nonetheless, all available evidence indicates that in complex, multifactorial RA models, capsaicin-sensitive afferents have predominantly anti-inflammatory effects. 

The increased early-phase spontaneous photon emission is also interesting from a technical standpoint, with several caveats. Our results show that even with sensitive imaging sequences increased photon count is barely detectable with the current commercial in vivo imaging systems. Administration of exogenous chemiluminescent sensors remains necessary in order to reliably assess ROS production in living subjects. The observed weak correlation between spontaneous photon emission and L-012 luminescence is in good agreement with prior results [26]. It also suggests that the overlap between these phenomena is partial, and spontaneous photon signal reflects different aspects of the inflammatory free radical burst. It should be noted that L-012 is activated in the presence of multiple free radicals released by both neutrophils and macrophages. Other luminescent probes such as luminol and lucigenin have been shown to correlate more selectively with neutrophil and macrophage activity respectively [31]. However, in order to detect ROS burst at the earliest possible time point, in the current study we have utilized a broad spectrum ROS sensor. 

The protective role of capsaicin-sensitive afferents in the K/BxN serum transfer arthritis model based on its profound effect on hyperacute vascular dysregulation and inflammatory cell activation is of great importance. Almost hundred years have passed since the first clinical, anecdotal observations suggesting the role of neuro-immune crosstalk in RA. Patients rendered hemiplegic by a prior stroke were observed to only develop arthritis on the functioning, non-paralyzed side. This clinical experience was recently corroborated by advanced in vivo imaging and transcriptomics methods, demonstrating that complete limb denervation inhibits the early, joint-localized vascular hyperpermeability in the K/BxN serum transfer model via modulating endothelial cell function [25]. However, denervation results in permanent injury to all motor, sympathetic, parasympathetic, and sensory fibers, thereby blurring the intricate functions of these neural elements, not to mention the consequent complete loss of limb function. In comparison, selective targeting of capsaicin-sensitive afferents to facilitate their anti-inflammatory mediator production and release without eliciting nociceptive signaling is technically feasible as a therapeutic approach as previously shown preclinically e.g., by tonic, low frequency stimulation of these sensory nerve terminals [32]. We have also shown previously that the absence of anti-inflammatory mediators released by capsaicin-sensitive afferents, such as galanin results in increased arthritis severity and vascular leakage in the K/BxN serum transfer model, while lack of vasoactive mediators such as PACAP diminishes vascular hyperpermeability [9,10]. Further work is therefore warranted to characterize the key early phase local mediators that are pivotal for the beneficial immune-regulatory effect of these afferents. 

## 4. Materials and Methods

### 4.1. Animals

Twelve- to sixteen-week-old male BALB/c were used for the experiment. The animals were bred at the SPF animal housing facility of the University of Pécs, and were subsequently kept at the Laboratory Animal House of the János Szentágothai Research Centre of the University of Pécs at 24–25 °C and provided with standard rodent chow and water ad libitum under 12h dark/12h light cycles.

The study was designed and conducted according to European legislation (Directive 2010/63/EU) and Hungarian Government regulation (40/2013., II. 14.) on the protection of animals used for scientific purposes. The project was approved by the Animal Welfare Committee of the University of Pécs, and the National Scientific Ethical Committee on Animal Experimentation of Hungary and licensed by the Government Office of Baranya County (license No. BA02/2000-28/2019, approved: 18 October 2019). All efforts were made to keep the number of experimental animals involved in the study at the necessary minimum.

### 4.2. Resiniferatoxin (RTX)-Induced Sensory Desensitization

Long-lasting defunctionalization of capsaicin-sensitive afferents was achieved by sc. treatment with high doses (10, 20, 70, and 100 µg/kg) of the ultrapotent capsaicin analogue RTX (Sigma Aldrich, St. Louis, MO, USA) on four consecutive days [33]. Mice also received simultaneously a solution containing 4% terbutaline-sulfate (AstraZeneca Ltd., Budapest, Hungary), 4% theophylline-ethylene diamin (Gedeon Richter Plc., Budapest, Hungary), and 2% athropine-sulfate (Egis Pharmaceuticals Plc., Budapest, Hungary) to decrease the systemic, mainly respiratory side effects of the RTX treatment. RTX was previously proved to be several magnitudes more potent TRPV1 receptor agonist than capsaicin, and it was also demonstrated that loss of vanilloid receptor expression in the central nervous system is not reversible [34,35]. After the desensitization has been completed the animals were left undisturbed for a period of two weeks, to limit the confounding effect of resultant stress, and also to allow for the systemic elimination of drugs used during the desensitization process. Desensitization was verified by the lack of eye-wiping behavior following 10 µL, 0.1 % capsaicin drop [22]. The experiment was commenced shortly after verifying the success of desensitization, in order to limit the potential biasing effects caused by peripheral recovery of vanilloid receptor expression. Non-desensitized age-matched mice were given the same treatment with the exception of RTX, and served as the control group. The capsaicin drop test was not used in the latter group. 

### 4.3. The K/BxN Serum Transfer Arthritis Model and the Experimental Layout

The sera of transgene positive (K/BxN) mice was obtained, pooled, and stored at −80 °C as described previously [36,37,38]. Arthritis was induced by ip. injection of 300 μL arthritogenic serum on day 0 of the experiment. In prior studies this was confirmed as a dose capable of eliciting robust joint inflammation, with minimal intra-group variation of disease severity [9]. 

Mice were monitored afterwards for 5 days with clinical scoring of arthritis severity, fluorescence imaging of the vascular leakage, and luminescence imaging of free radical production. Timing of fluorescent and luminescent imaging was different to reduce the possibility of pharmacological interaction and volume overload, and also due to the photosensitivity of the luminescent probe. On day 5 contrast-enhanced MRI of the ankle joints was performed. Thereafter the experiment was terminated, and mice were humanely euthanized by ip. sodium-pentobarbital. 

### 4.4. Clinical Arthritis Severity Scoring

Arthritis severity was assessed daily by clinical semiquantitative scoring of joint inflammatory changes (edema, hyperemia) on both hind limbs of the animals at 24 h intervals based on the hyperemia, and swelling of the ankle, footpad, and digits respectively (0–0.5 = intact limb, 10 = maximal swelling and hyperemia) throughout the experiment as described previously in details [9,38,39]. 

### 4.5. Assessment of Plasma Extravasation and Vascular Permeability

Plasma extravasation was evaluated using the near-infrared fluorophore IR-676 (0.5 mg/kg, Spectrum-Info Ltd., Kyiv, Ukraine) dissolved in 5% (*v*/*v*) aqueous solution of Kolliphor HS 15 (polyethylene-glycol-15-hydroxystearate; Sigma-Aldrich, St. Louis, MO, USA), yielding a micellar contrast agent accumulating in inflamed areas due to increased vascular permeability [40]. The formula was injected intravenously (iv.) into anesthetized mice through the retroorbital veins with a 0.5 mL insulin needle, the bevel oriented downwards as described earlier [41] 5 h after the K/BxN serum transfer and on day 2. Short-term anesthesia of multiple subjects has been done by administration of 120/6 mg/kg ketamine-xylazine ip. Fluorescence imaging of both ankle joints was performed in pairs 20 min post-injection using the IVIS Lumina III (PerkinElmer, Waltham, MA, USA) in vivo imaging system with the following settings: auto acquisition time, Binning = 2, F/stop = 2, excitation/emission filter: 660/710 nm). Data were analyzed and regions of interests (ROI) were drawn around the ankle joints. Fluorescence was expressed as total radiant efficiency ([photons/s/cm^2^/sr]/[μW/cm^2^]).

### 4.6. Evaluation of Free Radical Production

Free radical production by transmigrating neutrophils is an important early event in inflammatory arthritis, and we have previously shown that it correlates well with other (functional, histological) features of disease severity [9]. L-012 (Wako Pure Chemical Industries Ltd., Osaka, Japan) is a luminol analog chemiluminescent probe that has been shown to be very sensitive toward reactive oxygen and nitrogen species in vivo. L-012 was dissolved in sterile physiologic saline, and was injected in a dose of 25 mg/kg i.p. under as described previously [42] 3 h after K/BxN serum challenge, and on days 1 and 4 of the experiment. Mice were imaged in ketamine/xylazine anesthesia 10 min after the injection using the IVIS Lumina III system with the following settings: 120 s acquisition, Binning = 8, F/Stop = 1). Identical ROIs were applied around the ankle joints and chemiluminescence was expressed as total radiance (total photon flux/s).

### 4.7. Detection of In Vivo Spontaneous Ultraweak Photonic Emission

Prior to all further imaging anesthetized mice were imaged with the IVIS system using a dedicated imaging setting, with the aim to detect spontaneously emitted, extremely weak biophoton production resulting from singlet oxygen decay. For this purpose, a longer, 180 s luminescence acquisition was performed, with an increased binning factor of 16. To exclude the bias effect of body heat changes the imaging chamber of the IVIS system was maintained at 37 °C throughout the experiment. 

### 4.8. In Vivo Contrast Enhanced MRI 

In vivo contrast-enhanced MRI has been previously demonstrated to be suitable to monitor and characterize joint damage in the K/BxN serum transfer model [43]. Imaging was performed on day 5 of the experiment. Mice were anesthetized with 3% isoflurane, and were transferred into the Bruker PharmaScan 4.7T (Bruker, Billerica, MA, USA) imaging system. Because of the repeated acquisitions pre- and postcontrast adequate anesthesia was maintained by further isoflurane inhalation through nosecone, while respiratory rate was constantly monitored to control the depth of anesthesia. Mice were positioned on their back, and the right ankle joint was then secured in a flexed position using a custom-made styrofoam support and plastic strips. First a noncontrast 2D multislice T1 RARE (TR: 1200 ms, TE: 8.3 ms) sequence had been performed in the saggital plane, then the mice were briefly removed from the system, and while still under anesthesia 100 µL of the gadolinium-based, clinically approved MRI contrast agent gadobutrol (Gadovist^®^, Bayer Schering Pharma AG, Berlin, Germany) solution (1 mmol/mL) was injected i.v. via the retro-orbital venous plexus. The mice were immediately transferred back to the system afterwards, and the same sequence was repeated 10 min post-injection. Total acquisition time of each sequence was approximately 45 min per animal. Images were reconstructed using the Bruker ParaVision™ 6.0.1 software. The scans were exported in the DICOM format, and ROI-based quantification was performed with the RadiAnt™ 2020.2 software [44]. In each scan ROIs have been applied to all tarsal and tibiotarsal joints clearly depicted in the imaging plane, and the peak arbitrary signal value was used for further assessment. Values from at least 3–5 joints/subject have been averaged, and this mean was used for further comparison. 

### 4.9. Statistics

The clinical severity, fluorescent and luminescent optical imaging results were analyzed using two-way ANOVA and the Fishers LSD test. Correlation between imaging data and clinical severity was determined with linear regression and correlation. The intra-articular enhancement on MRI was assessed with unpaired t-test. All analyses had been performed in the GraphPad Prism™ 7.0 statistical software. All error bars represent the standard errors of means (SEM), at least *p* < 0.05 was considered to be statistically significant. Because of the small sample size no formal normality test was conducted. 

## 5. Conclusions

The present results provide the first evidence for pivotal protective roles of capsaicin-sensitive afferents in the hyperacute phase of inflammatory arthritis. Their activity reduces vascular leakage. This is also coupled with diminished leukocyte ROS burst, which can be an indirect outcome of the vascular changes and consequently decreased ability to transmigrate into the inflamed joint tissues, and/or also due to direct inhibitory action via local mediators. The above parameters show correlation with each other and the disease severity observed later on. Furthermore, optical imaging methods provide sensitive, specific, and reliable tools to investigate the mechanisms related to the very early inflammatory phase, which is not possible with conventional functional and morphological investigational techniques. 

## Data Availability

The data presented in this study are available on request from the corresponding author.
